# Quality by design paradigm for optimization of green stability indicating HPLC method for concomitant determination of fluorescein and benoxinate

**DOI:** 10.1038/s41598-023-37548-5

**Published:** 2023-06-28

**Authors:** Amira H. Kamal, Ahmed A. Habib, Sherin F. Hammad, Safa M. Megahed

**Affiliations:** Department of Pharmaceutical Analytical Chemistry, Faculty of Pharmacy, Tanta University, Tanta, Egypt

**Keywords:** Chemistry, Analytical chemistry

## Abstract

A green, robust and fast stability indicating chromatographic method has been developed for concomitant analysis of fluorescein sodium and benoxinate hydrochloride in the presence of their degradation products within four minutes. Two different designs including fractional factorial and Box–Behnken designs were implemented for screening and optimization steps, respectively. The optimum chromatographic analysis was achieved using a mixture of isopropanol and 20 mM potassium dihydrogen phosphate solution (pH 3.0) in the ratio 27:73 as mobile phase. The flow rate was 1.5 mL/min and column oven temperature was 40 °C. Chromatographic analysis was performed on Eclipse plus C18 (100 mm × 4.6 mm × 3.5 μm) column with DAD detector set at 220 nm. A linear response was acquired over the range of 2.5–60 μg/mL and 1–50 μg/mL for benoxinate and fluorescein respectively. Stress degradation studies were executed under acidic, basic, and oxidative stress conditions. The method was implemented for quantitation of cited drugs in ophthalmic solution with mean percent recovery ± SD of 99.21 ± 0.74 and 99.88 ± 0.58 for benoxinate and fluorescein respectively. The proposed method is more rapid and eco-friendly compared to the reported chromatographic methods for determination of cited drugs.

## Introduction

Nowadays, there is growing interest in development of environment-friendly analytical methods that replace the highly toxic solvents with more eco-friendly ones and reduce the amount of organic solvents used. Different tools have been proposed for assessment of analytical procedures greenness^[1–3]^. Among those tools, two quantitative tools are available including analytical eco-scale, and HPLC-EAT (environmental assessment tool). Analytical eco-scale assesses greenness depending on penalty points assigned for each reagent and instrument used^[4]^. From the total of 100 points, penalty points are subtracted for each negative effect on the environment, such as hazardous chemicals used, waste generation and high energy consumption. Analytical eco-scale tool has many advantages such as the ability to calculate the amounts of chemicals and waste quantitatively. Moreover, it allows comparison of different analytical methods regarding greenness easily. However, there is one main drawback of this assessment tool that the final score is not informative of the cause of negative environmental effect.

HPLC-EAT (environmental assessment tool) is a specific software dedicated to the assessment of chromatographic methodologies regarding greenness based on mobile phase composition as it focuses on solvent amount and type^[5]^. It is calculated based on the sum of safety, health, and environmental factors for a given amount of solvent applied during chromatographic analysis, thus reflects the overall greenness of the method. The lower the EAT value, the greener the analytical method. The advantage of this tool for assessment is that it is a simple method with a free software. Moreover, it can be used for rapid comparison between different chromatographic runs. However, its application is limited to chromatographic techniques only.

A new tool has been introduced for greenness evaluation of analytical procedures based on principles derived from the twelve principles of green analytical chemistry called AGREE^[6]^. The AGREE software generates a clock-like outline with numbers 1–12 around the edge, indicating the 12 principles of green analytical chemistry. Another recently introduced greenness assessment tool is complexGAPI which represent method greenness in the form of colored pictograms^[7]^. The application of ComplexGAPI is based on simple free software. It assigns the greenness of each step of an analytical methodology using a pictogram and color scale with three levels of assessment corresponding to different levels of impact on the environment, with low, medium and high levels corresponding to green, yellow and red, respectively.

Recently, implementation of principles of Quality by Design (QbD) in various pharmaceutical aspects is gaining interest^[8–10]^. The implementation of QbD paradigm to analytical procedure development emphasizes the concept of establishing quality during method development, rather than testing method’s quality after development. QbD paradigm follows a systematic path in method development thus ensures method’s robustness.

First, the goal of the method to be developed, which is called Analytical Target Profile (ATP), is defined and then critical method parameters (CMPs) that have impact on critical quality attributes (CQAs) are assigned.

Various experimental designs have been performed during validation, development, and optimization of different analytical procedures^[11–17]^. Among available designs fractional factorial (FFD) design is commonly applied in screening of large number of factors with few number of experiments. Box Behnken Design (BBD) is used to attain optimum conditions by studying the parameters at three levels with a fewer experiments compared to other three level designs such as central composite and three level full factorial designs^[18]^.

The chemical analysis of ophthalmic dosage forms is challenging because they contain a large number of excipients to regulate osmotic pressure, the pH, and viscosity of the preparation. They may also contain preservatives especially in multiuse package^[19]^.

**Figure 1:**
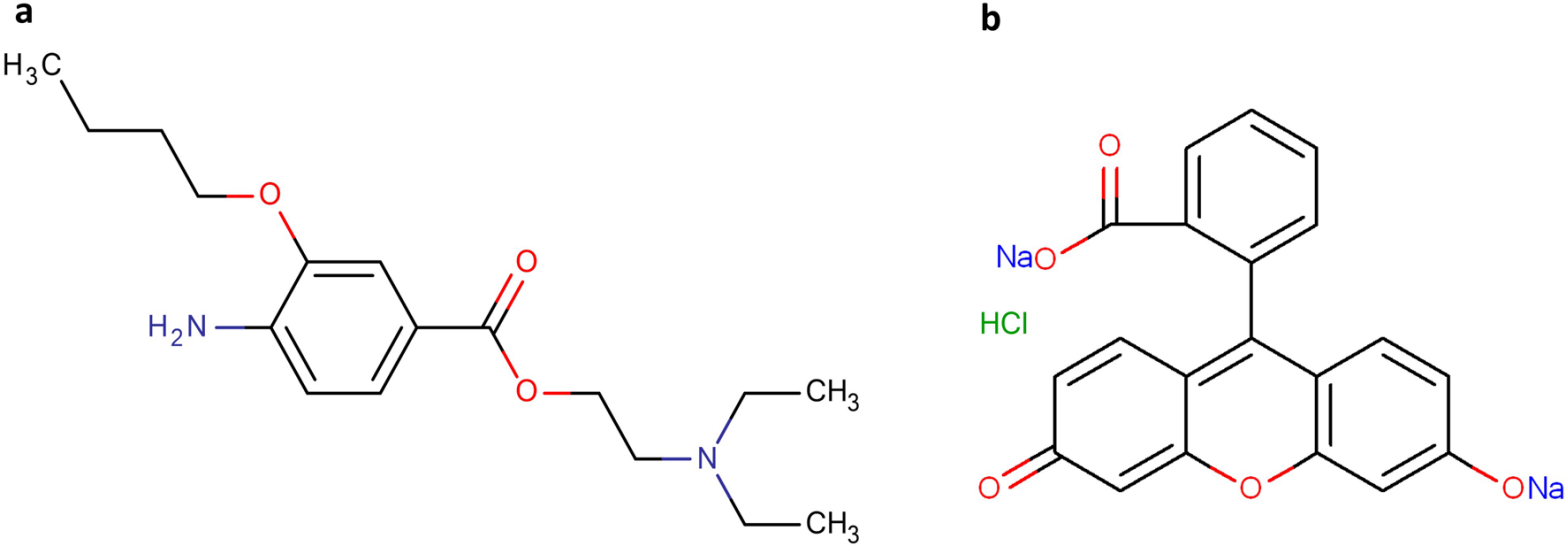
Chemical structure of (**a**) BNX HCl, and (**b**) FLR sodium.

Benoxinate hydrochloride (BNX HCl) is used as topical anaesthesia for the eye in minor surgery. Fluorescein sodium (FLR sodium) is water soluble fluorescent dye which is used as a diagnostic agent in corneal injuries and corneal trauma. Recently, FDA has approved ophthalmic solution containing FLR sodium (0.25%) and BNX HCl (0.40%) for procedures that requires a disclosing agent together with an anesthetic agent such as removal of foreign bodies from cornea and other short conjunctival or corneal surgeries^[20]^. Chemical structures of both drugs are shown in Fig. 1.

Only few number of analytical methods has been reported for concomitant assay of BNX HCl and FLR sodium including UV spectrophotometric methods^[21]^, and chromatographic methods^[22,23]^. However, both reported chromatographic methods involve the use of acetonitrile as organic modifier which cause harm to health and environment. Therefore, there is a need for developing green eco-friendly stability-indicating method for rapid determination of cited drugs in the presence of their degradation products.

The aim of the current study was to develop green stability-indicating method for concomitant assay of BNX HCl and FLR sodium in the presence of their degradation products, by replacing the conventional harmful reagents and solvents used in chromatographic analysis with more environment-friendly ones in addition to minimizing analysis time to reduce amount of waste. It involved implementation of designs of experiment for screening and optimization of all chromatographic factors. The greenness of the method was taken into consideration in the development of the proposed method from the first step by assigning ecoscale and EAT scores as responses to be optimized throughout method development. The developed method is the first green chromatographic method for concomitant determination of FLR sodium and BNX HCl.

## Materials and methods

### Materials and reagents

All chemicals utilized throughout the present work were of analytical grade.

BNX HCl powder (99.80%) was provided as a gift from EIPICO company (10th of Ramadan, Egypt). FLR sodium (99.20%) was kindly supplied by Sigma Pharmaceutical Industries (Quesna, Egypt).

HPLC grade solvents as ethanol (Fisher scientific, Germany), and isopropanol (Hipersolv, BDH Laboratory supplies, England) as well as analytical grade ortho-phosphoric acid (Ridel-de Haën, Germany), sodium Hydroxide sodium (WinLab, UK), hydrochloric acid (Ridel-de Haën, Germany), and potassium dihydrogen phosphate (Fisher scientific, Germany) were used.

### Instrument and software

Chromatographic analysis was accomplished using Dionex Ulti-Mate 3000 RS system (Thermo Scientific, Dionex, USA), composed of Quaternary RS pump, RS auto-sampler injector, Thermo-stated RS column compartment, and RS PDA detector. A Dell computer was connected to the HPLC instrument, provided with Chromeleon 7.1 edition software.

HANNA pH 211 Micro-processor pHmeter was used for pH measurements. Forced degradation studies were accomplished with the aid of thermostatic controlled water bath (Memmert, Germany).

Experimental design analysis including matrix of fractional factorial (FFD) and Box–Behnken (BBD) designs were executed using Design Expert software (Stat_Ease Inc. Minneapolis, USA); version 11.1.2.0.

### Chromatographic conditions

Separation was accomplished on Eclipse plus C18 (100 mm × 4.6 mm × 3.5 μm) column with controlled pore size of 95A using a mixture of 27% (v/v) isopropanol and 73% (v/v) 20 mM potassium dihydrogen phosphate buffer (pH adjusted to 3.0) as mobile phase at a flow rate of 1.5 mL/min. Column temperature was set at 40 °C The detection was performed at 220 nm using DAD detector.

### Preparation of stock standard solutions

Stock standard solutions of BNX HCl (1 mg/mL) was prepared dissolving 50 mg of BNX HCl powder in distilled water in 50-mL volumetric flask. Similarly, Stock standard solutions of FLR sodium (1 mg/mL) was prepared using distilled water.

### Construction of calibration curves

Into a group of 10-mL volumetric flasks, aliquots of FLR sodium and BNX HCl stock standard solutions were quantitatively transferred; appropriate dilutions were carried out with mobile phase to yield solutions in the concentration range of 2.5–60.0 μg/mL for BNX HCl and 1.0–50.0 μg/mL for FLR sodium. Portions of each solution (injection volume of 20 µL) were injected in triplicates under the mentioned chromatographic conditions.

### Forced degradation studies

BNX HCl and FLR sodium mixture was subjected to the stress conditions shown in the reported method^[22]^ as following:

#### Acidic degradation

Acidic degradation studies were executed by weighing 16 mg of BNX HCl and 10 mg of FLR sodium, dissolving in 0.75 N HCl in 10-mL volumetric flask and heating for one hour at 75 °C.

#### Alkaline degradation

Alkaline degradation studies were executed by weighing 16 mg of BNX HCl and 10 mg of FLR sodium, dissolving in 0.03 N NaOH in 10-mL measuring flask and heating for one hour at 25 °C.

#### Oxidative degradation

Oxidative degradation studies were executed by weighing 16 mg of BNX HCl and 10 mg of FLR sodium, dissolving in 10% hydrogen peroxide in 10-mL volumetric flask and heating under reflux for six hours at 50 °C.

250 µL aliquots of each solution were transferred into 10-mL measuring flasks, cooled and neutralized (in case of acidic and alkaline degradation), then completed to the mark with the mobile phase.

### Analysis of synthetic mixture

Since eye drops containing BNX HCl and FLR sodium mixture is not available in the local Egyptian market, synthetic mixture was formulated to simulate the dosage form. Synthetic mixture was prepared by weighing 25 mg FLR sodium, 40 mg BNX HCl, 100 mg chlorobutol, 100 mg boric acid and 1 gm povidone, and dissolving them with distilled water into 25-mL measuring flask.

A number of aliquots of this solution (each aliquot equals 250 µL) were transferred into a set of 10-mL measuring flask, completing to the mark with mobile phase to get solutions containing 40 µg/mL BNX HCL and 25 µg/mL FLR sodium. A volume of these final solutions equals 20 µL was injected in triplicate in the chromatographic system under the specified conditions. The concentration of each drug in assay solutions was found using the corresponding regression equation.

## Results and discussion

### Method development and optimization

Quality by Design paradigm was implemented throughout method development. First, the analytical target profile; ATP, which is the purpose of the method is assigned. The purpose was development of fast green stability indicating HPLC method for determination of BNX and FLR in the presence of their stress degradation products. Then, CQAs (critical quality attributes) are defined including short analysis time, good peak symmetry, increased number of theoretical plates and the method greenness. The method greenness was taken as one of the responses to be optimized from the first step in screening phase in order to get the best chromatographic conditions not only regarding analytical performance but regarding greenness as well. Then, preliminary experiments were carried out to identify CMPs (critical method parameters) which could affect CQAs.

#### Screening of parameters

The traditional trial and error method for optimization of chromatographic conditions is tedious due to large number of factors to be studied. Fractional factorial (FFD) design was selected for screening phase as it is the most convenient design for studying a large number of factors. FFD was selected for screening of eight factors which are pH of aqueous portion of the mobile phase, buffer concentration, organic modifier, ratio of organic modifier, triethylamine concentration, column temperature and flow rate. The low and high levels of each factor were defined.

A number of sixteen experimental runs were executed as presented in the design matrix in supplementary material (Table S1). Different responses including resolution between BNX and FLR peaks, tailing of peaks, run time, analytical eco-scale and EAT score were recorded. Both analytical eco-scale and EAT score were calculated for each run as indicative tools for method greenness. Eco-scale alone does not give good indication for method greenness as it does not adequately reflect the differences in the amount of organic solvents consumed in different runs. Therefore, EAT score was used in addition to eco-scale as responses that reflect method greenness.

#### Fractional factorial design results

**Figure 2:**
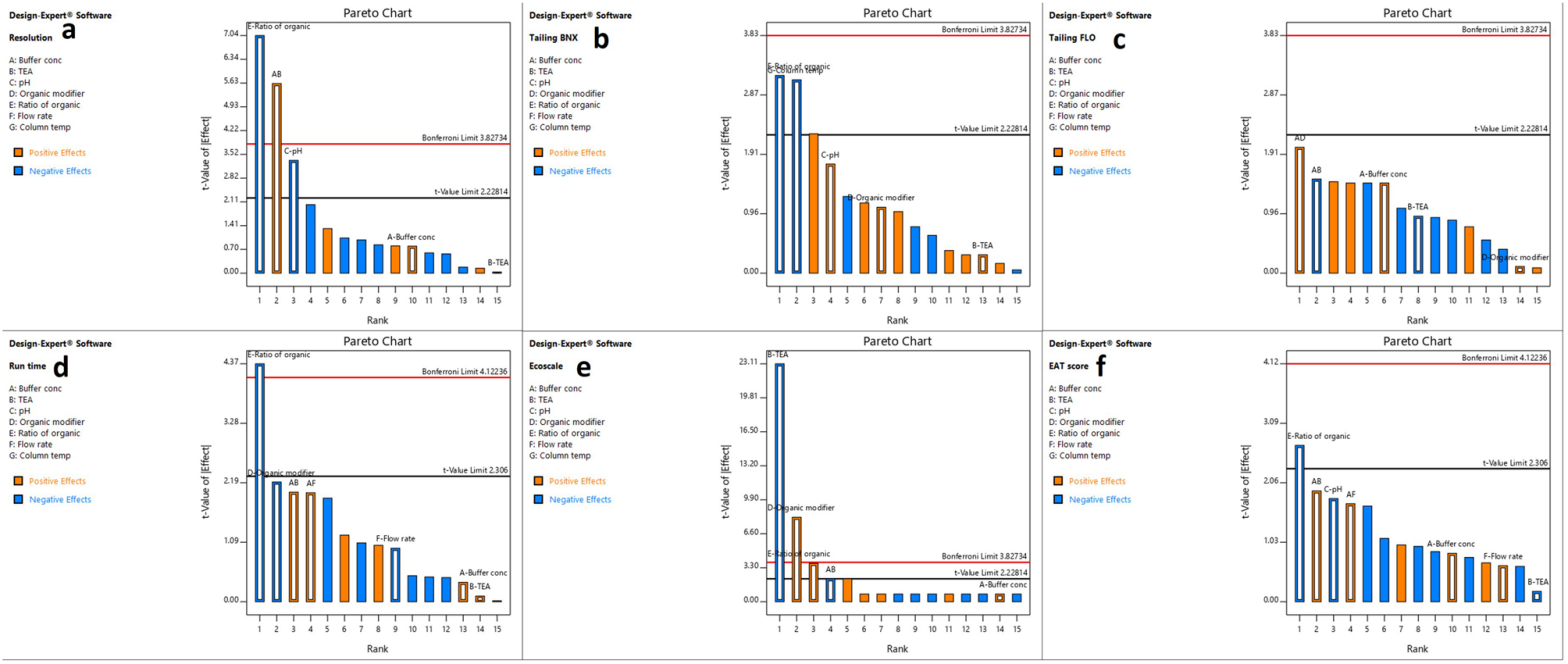
Pareto charts of the effects on the chromatographic responses: (**a**) resolution, (**b**) tailing of BNX, (**c**) tailing of FLR, (**d**) run time, (**e**) analytical ecoscale score, and (**f**) EAT score.

The most statistically significant factors that greatly affect different responses were identified using pareto charts. Significant factors are those which exceed t-limit line. Figure 2 presents Pareto charts which show that the ratio of organic modifier has significant effect on resolution between BNX and FLR peaks, tailing of BNX peak, and run time meanwhile pH significantly affects resolution. Column temperature has significant effect on tailing of BNX peak. Flow rate and buffer concentration did not have any significant impact on any response. Isopropanol was found to be better than ethanol regarding run time and method greenness. Triethylamine had negative effect on eco-scale and it did not significantly affect any other CQA, therefore it was removed from mobile phase to improve greenness of the method.

From the pareto charts results, three factors which have the greatest significance were selected for further optimization including ratio of organic modifier, pH of phosphate buffer, and column temperature. Other factors were set at their values that result in better method greenness and chromatographic performance (organic modifier isopropanol, buffer concentration 20 mM, flow rate 1.5 mL/min, without triethylamine).

#### Optimization using Box Behnken design

For optimization of the crucial factors, Box Behnken (BBD) design was applied as it requires less number of experimental runs to get quadratic model compared to other experimental designs such as Central Composite (CCD) Design. Since the aim of this work is the development of stability-indicating method, the resolution between drug peaks and the peaks of different degradation products were taken as responses to be optimized using BBD. Responses included resolution 1 (resolution between BNX and FLR peaks), resolution 2 (resolution between FLR peak and peak of BNX hydrolysis degradation product), resolution 3 (resolution between BNX and its oxidative degradation product peaks), resolution 4 (resolution between FLR and its oxidative degradation product peaks), tailing of BNX and FLR peaks, run time, and EAT score as an indicator for method greenness.

A set of 15 experimental runs were executed and responses were recorded as presented in supplementary material (Table S2). Isopropanol was the organic solvent used for all experimental runs in this optimization phase, this led to the same value of eco-scale in all experiments , in contrast with EAT score. Therefore, only EAT score was used as indicative tool of greenness during optimization step.

#### Box Behnken design results

The data obtained from the fifteen runs was analysed by the aid of Design Expert software, and various plots were generated. Three dimensional response surface plots show how the response changes depending on the factors values and are used to find the optimal conditions. A color code is associated with each plot to allow prediction of the response’s value. Blue color represents the low value whereas red color indicates greater value of a response. 3D surface plots in supplementary material (Figs. S1 and S2) illustrated the impact of ratio of organic modifier and pH on different responses. From these plots, increasing ratio of organic modifier is associated with decreasing resolution-1, resolution-3 and resolution-4 at lower pH values. On contrast, resolution-2 increases at middle values of organic modifier ratio at lower pH values. Accepted low values of tailing of both peaks, run time, and EAT scores are acquired at low pH and organic modifier ratio.

The ultimate goal of optimization phase is to find the optimum conditions that give the best response values. This is accomplished using a desirability function D that has values range between d = zero (for an unacceptable response value) and d = one (for a completely desirable one).

Individual desirability values are calculated for each single response, then the overall desirability, D, is calculated by combining these individual desirability values.

**Figure 3:**
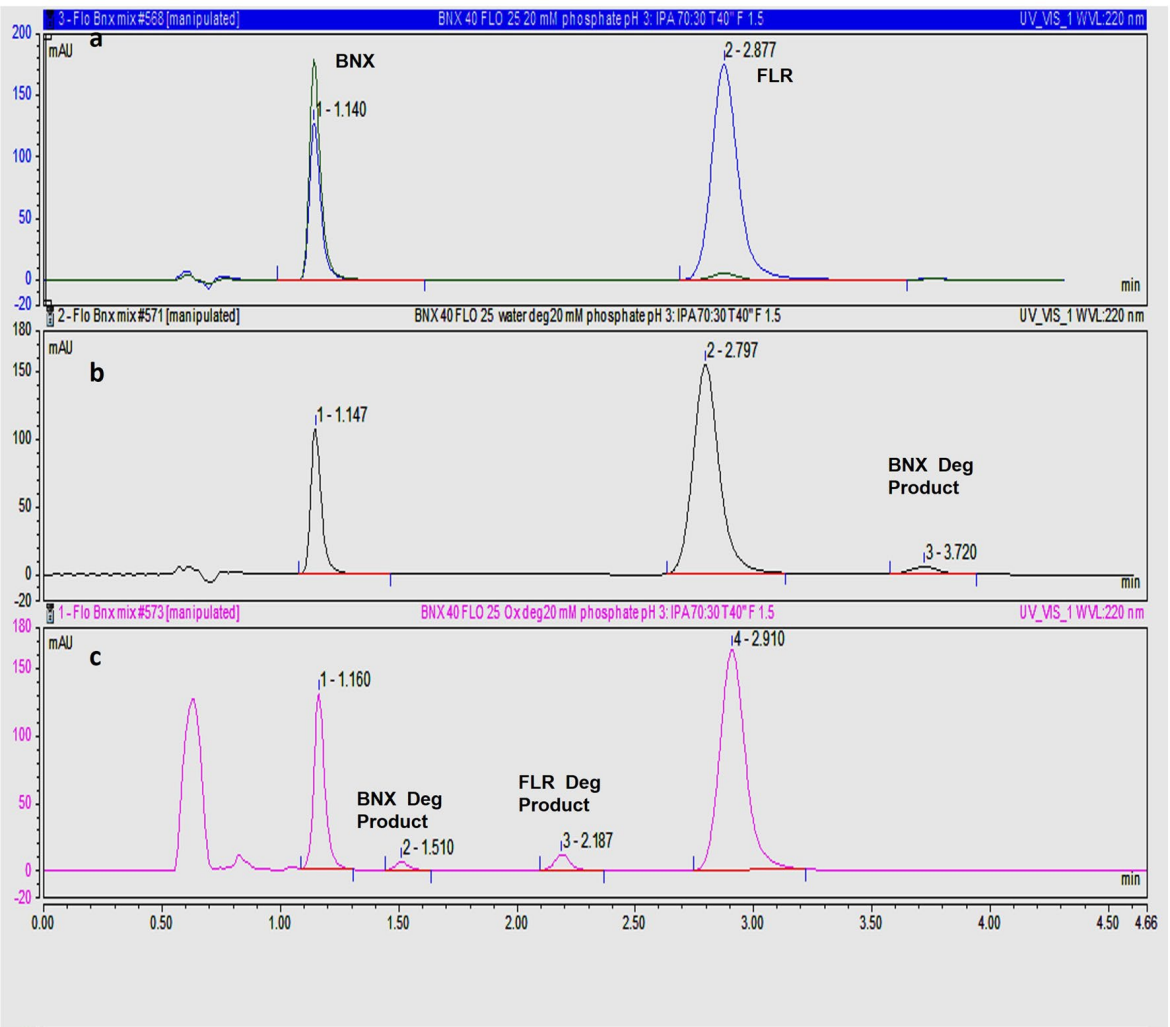
HPLC Chromatogram of (**a**) BNX 40 µg/mL and FLR 25 µg/mL, (**b**) acid degraded sample, and (**c**) oxidative degraded sample.

Desirability plot helped to identify the optimum chromatographic conditions for the proposed stability indicating chromatographic method as presented in supplementary material (Fig. S3). The optimum conditions involved the use of mobile phase consisting of a mixture of isopropanol and 20 mM potassium dihydrogen phosphate solution (pH adjusted to 3.0) in the ratio 27:73 at a flow rate of 1.5 mL/min and column oven temperature of 40 °C. Chromatogram of a binary mixture containing 25 µg/mL FLR sodium and 40 µg/mL BNX HCl under the previously mentioned chromatographic conditions is shown in Fig. 3a. Parameters of system suitability of the developed chromatographic method are listed in supplementary material (Table S3).

#### Design space deduction

The design space provides the range of interactions between crucial factors and their impact on different responses that had been studied during optimization step. It assures developed method’s quality. Regions that are shaded out in grey represents the range of factors where the specifications of a given response were not met, while the yellow region represents a favorable region at which desired responses are full filled as shown in supplementary material (Fig. S4). In other words, within the yellow regions in design space the method is robust and the quality of the proposed method will not be affected by changes made within this region.

#### Forced degradation studies

Forced degradation studies were executed under acidic, alkaline and oxidative stress conditions. Details of degradation results including degradation conditions, percent degradation, and retention time of degradation products were listed in Table 1.The chromatographic method was successfully applied to different stressed samples with good resolution between peaks of drugs and degradation products peaks. HPLC Chromatograms of BNX HCl and FLR sodium under different stress conditions are shown in Fig. 3.

**Table 1:** Degradation results of BNX HCl and FLR sodium.

Stress condition	Degradation conditions	% Degradation (BNX HCl)	% Degradation (FLR sodium)	Retention time of degradation product (minute)
Acidic degradation	0.75 N hydrochloric acid for 1 h at 75 °C	23.00%	No significant degradation	3.37
Basic degradation	0.03 N sodium hydroxide for 1 h at 25 °C	19.00%	No significant degradation	3.39
Oxidative degradation	10% H2O2 for 6 h at 50 °C	20.00%	18.00%	BNX degradation product at 1.50
				FLR degradation product at 2.19

### Method validation

The developed analytical procedure was validated in compliance with the ICH guidelines^[24]^.

#### Linearity and range

The linearity of BNX HCl and FLR sodium was confirmed by construction of the calibration curves in the range of 2.5–60 µg/mL and 1–50 µg/mL respectively. Calibration curves were acquired by plotting mean peak area versus concentration of each drug. The correlation coefficients values (0.9998 for both drugs) indicated the good linearity. Linearity regression parameters for BNX HCl and FLR sodium using the proposed HPLC method are presented in Table 2.

**Table 2:** Linearity regression parameters for BNX HCl and FLR sodium using the proposed HPLC method.

Parameter	BNX HCl	FLR sodium
Linearity range (µg/mL)	2.50–60.00	1.00–50.00
slope	0.26	1.15
SE of slope	0.001	0.006
Intercept	− 0.31	0.15
SE of intercept	0.04	0.14
Correlation coefficient (r)	0.9998	0.9998
SE of estimation	0.08	0.28

*SE* standard error.

#### Limits of quantitation (LOQ) and detection (LOD)

The ICH (Q2 (R1)) guidelines were implemented for the determination of LOQ and LOD based on the standard deviation of the blank response and the slope of the calibration curve. For BNX HCl, the values of LOD and LOQ were 0.46 and 1.40 µg/mL, while for FLR sodium, they were 0.25 and 0.75 µg/mL.

#### Accuracy and precision

Accuracy of the developed method was assessed by calculating the mean percent recoveries of triplicate determination for BNX HCl and FLR sodium at three concentrations within the linearity range as shown as shown in supplementary material (Table S4).

The good % recovery values indicate the accuracy of the developed method. Repeatability and intermediate precision (intraday and inter-day precisions) were assessed by determining the values of the standard deviation and % relative standard deviation for triplicate assays of three concentrations of BNX HCl and FLR sodium within the linearity range in the same day and on three different days respectively. % RSD values did not exceed 2% as shown in supplementary material (Table S5) which confirmed the precision of the proposed method.

#### Robustness

Robustness of the developed methodology was evaluated by making small variations within its optimized conditions. The parameters which were examined in the robustness testing included the change in pH (3.0 ± 0.1), ratio of isopropanol (27 ± 2%), column temperature (40 °C ± 2), and flow rate (1.5 mL/min ± 0.1). RSD values were less than 2% indicating robustness of the method as shown in supplementary material (Table S6).

The implementation of experimental designs resulted in development of a robust stability indicating chromatographic method for analysis of both drugs within reasonable time.

### Application to formulated eye drops

The developed method was implemented for the quantitation of BNX HCl and FLR sodium in their laboratory formulated eye drops with no interference from excipients. Satisfactory results were obtained for both drugs as shown in supplementary material (Table S7). The results of the proposed method were compared with that of the reference method^[22]^. According to the findings of the students’ t-test and F-test, which are displayed in the shown in supplementary material (Table S7), there was no significant difference between the adopted method and the reference one. The chromatogram of laboratory formulated eye drops is shown in supplementary material (Fig. S5).

### Greenness assessment of the developed method

**Figure 4:**
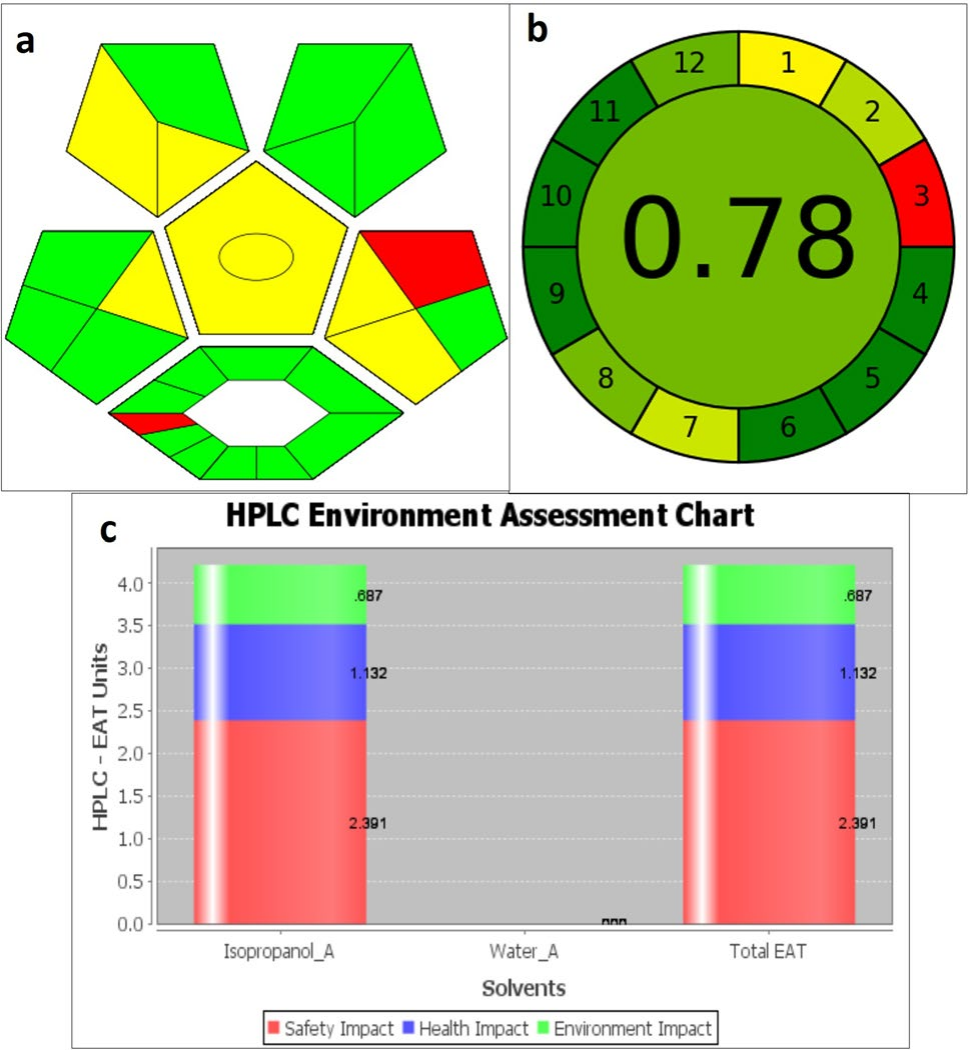
Results of greenness evaluation of the developed method using (**a**) complexGAPI, (**b**) AGREE tool, and (**c**) HPLC-EAT score.

The proposed chromatographic method’s greenness of was proved by the high value of calculated analytical eco-scale score (90) as shown in Table 3. In addition, the low value of EAT score (4.21) indicated that the proposed method is green. The method was also proved to be green as evaluated by the newly introduced tools AGREE and complexGAPI. Figure 4 presents results of greenness assessment for the developed method. The proposed method was found to be more rapid and eco-friendly than reported ones as shown in Table 4.

**Table 3:** Calculation of analytical eco-scale for the developed method.

Reagents/instruments	Penalty points
Reagents	
Phosphoric acid	2
Isopropanol	4
Instruments	
HPLC	1
Occupational hazards	0
Waste	3
Total penalty points	∑10
Analytical eco-scale score	90

**Table 4:** Comparison of the proposed method and reported methods for determination of BNX HCl and FLR sodium.

	Reported method^[22]^	Reported method^[23]^	Proposed method
Stationary phase	Inertsil ODS (250 mm × 4.6 mm, 5 μm) column	4 mm × 30 cm column that contains packing L1	Eclipse plus C18 (100 mm × 4.6 mm × 3.5 μm) column
Mobile phase	Acetonitrile and 50 mM potassium dihydrogen phosphate buffer containing 0.01% triethylamine (pH adjusted to 5.0) in a ratio 40:60 v/v	Sodium 1-pentanesulfonate dissolved in glacial acetic acid (40%) and acetonitrile containing triethanolamine (60%) and pH is adjusted to 3.0 with phosphoric acid	Mixture of 27% (v/v) isopropanol and 73% (v/v) 20 mM potassium dihydrogen phosphate buffer (pH adjusted to 3.0)
Run time	8 min	19 min	4 min
Detection wavelength	220 nm	254 nm	220 nm
Linearity range (µg/mL)	4.0–80.0 for BNX 1.0–50.0 for FLR	NA	2.5–60.0 for BNX 1.0–50.0 for FLR
Analytical eco-scale score	81	81	90

## Conclusion

A green, simple, and rapid stability-indicating chromatographic method was developed for the concomitant determination of BNX HCl and FLR sodium in their bulk and ophthalmic solution within four minutes. The adopted method was successfully applied to different stress degraded samples of both drugs without interference from excipients or degradation products. Experimental design paradigm was implemented throughout the development of the method. Combining experimental designs with green analytical chemistry results in the development of a design space that ensures the method's quality and improves its robustness. The proposed method can be applied for simultaneous quantitation of cited drugs in eye drops. It can be extended for their determination in aqueous humor. Being eco-friendly and fast, the adopted method can replace the reported HPLC methods for analysis of both drugs in routine analysis.

## Supplementary Information

Supplementary Information.

## Data Availability

The datasets generated and/or analyzed during this study are included in the article and supplementary material. More data are available from the corresponding author on reasonable request.
