# Supplementary material for: Quality by design paradigm for optimization of green stability indicating HPLC method for concomitant determination of fluorescein and benoxinate
Source: Sci Rep. 2023 Jun 28;13:10471. doi: 10.1038/s41598-023-37548-5 (PMC10307890; doi:10.1038/s41598-023-37548-5)
Supplement: Supplementary file 1 — Supplementary Information. [file 41598_2023_37548_MOESM1_ESM.docx]

**Quality by Design Paradigm for Optimization of Green Stability Indicating HPLC Method for Concomitant Determination of Fluorescein and Benoxinate**

**Amira H. Kamal^1^, Ahmed A Habib^1^, Sherin F Hammad^1^, and Safa M Megahed^1^***

^1^ Department of pharmaceutical analytical chemistry, Faculty of Pharmacy, Tanta University, Tanta, Egypt.

*Corresponding author

e-mail: [safa.megahed@gmail.com](mailto:safa.megahed@gmail.com), safa.megahed.pharm.edu.tanta.eg

Supplementary material

**Figure Caption:**

**Supplementary Fig. S1:** Three dimensional plot of the effects on the chromatographic responses: resolution 1 (between BNX and FLR peaks), resolution 2 (between FLR peak and peak of BNX hydrolysis degradation product), resolution 3 (between BNX and its oxidative degradation product peaks), and resolution 4 (between FLR and its oxidative degradation product peaks).

**Supplementary Fig. S2:** Three dimensional plot of the effects on the chromatographic responses: tailing BNX peak, tailing FLR peak, run time, and EAT score.

**Supplementary Fig. S3:** Desirability plot showing optimum chromatographic conditions.

**Supplementary Fig. S4:** Design space (overlay plot) for different chromatographic conditions.

**Supplementary Fig. S5:** Chromatograms of laboratory formulated eye drops containing 40 µg/mL BNX HCl and 25 µg/mL FLR sodium using the proposed HPLC method.


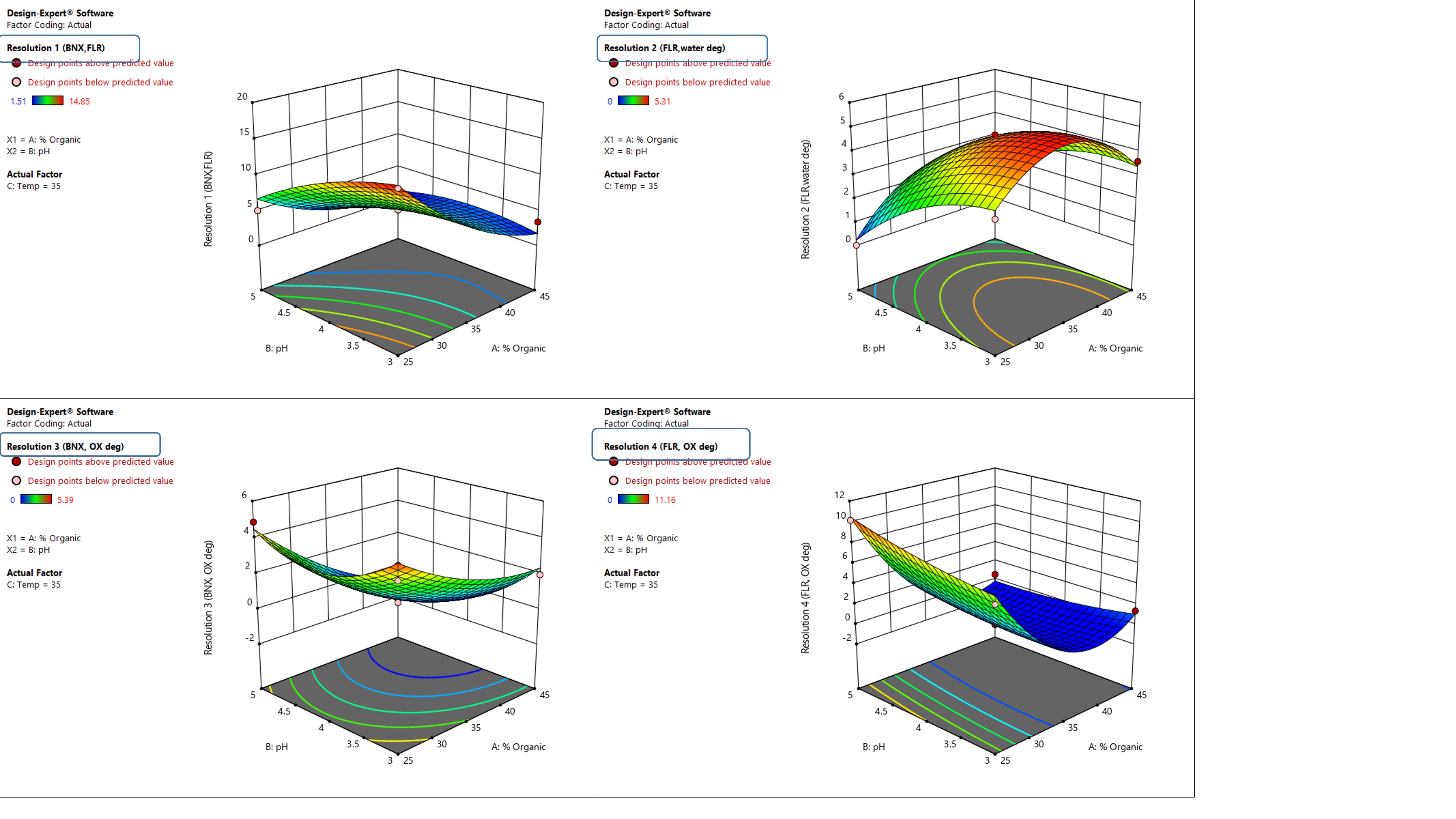


**Supplementary Fig. S1**


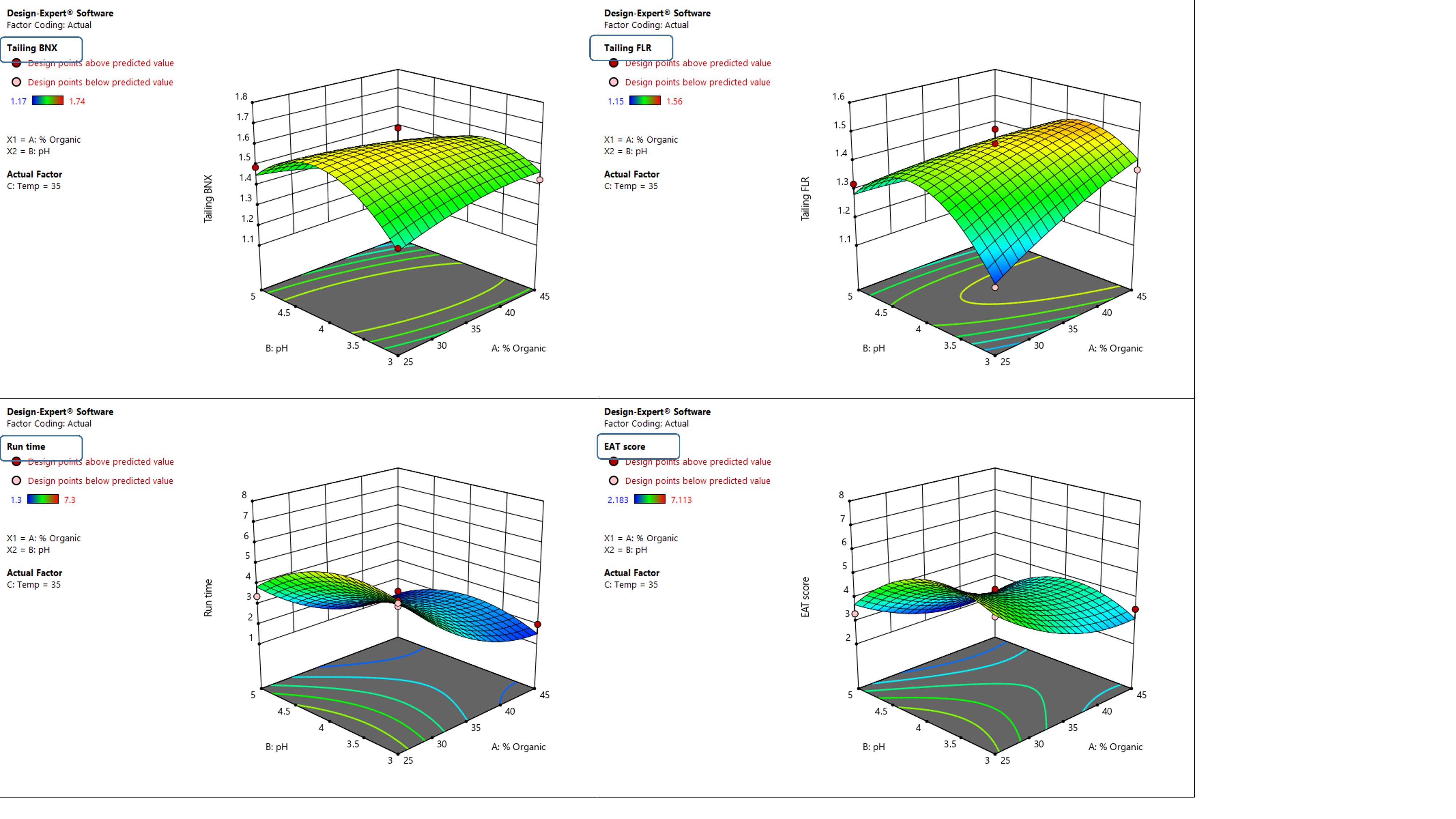


**Supplementary Fig. S2**


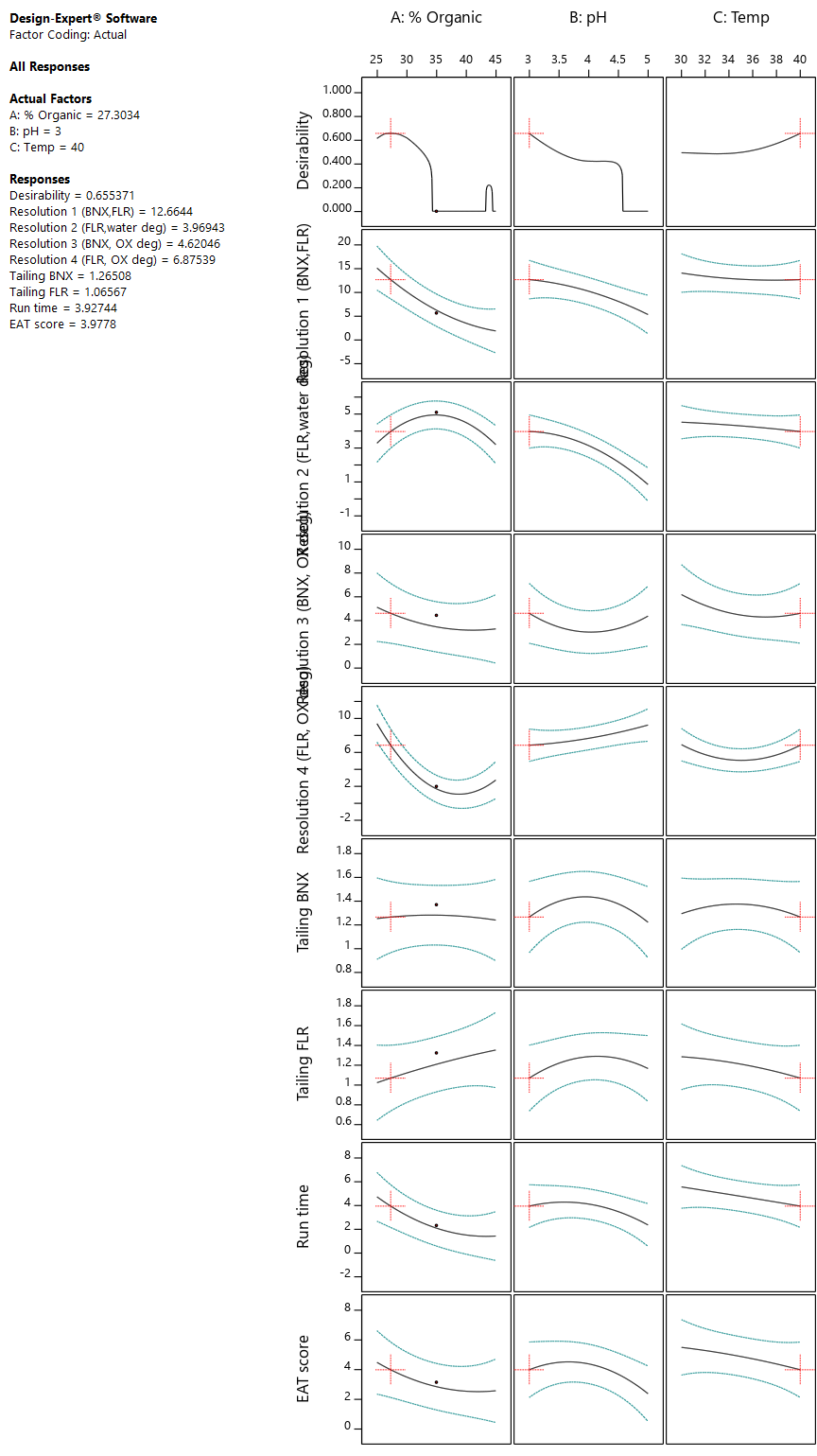


**Supplementary Fig. S3**


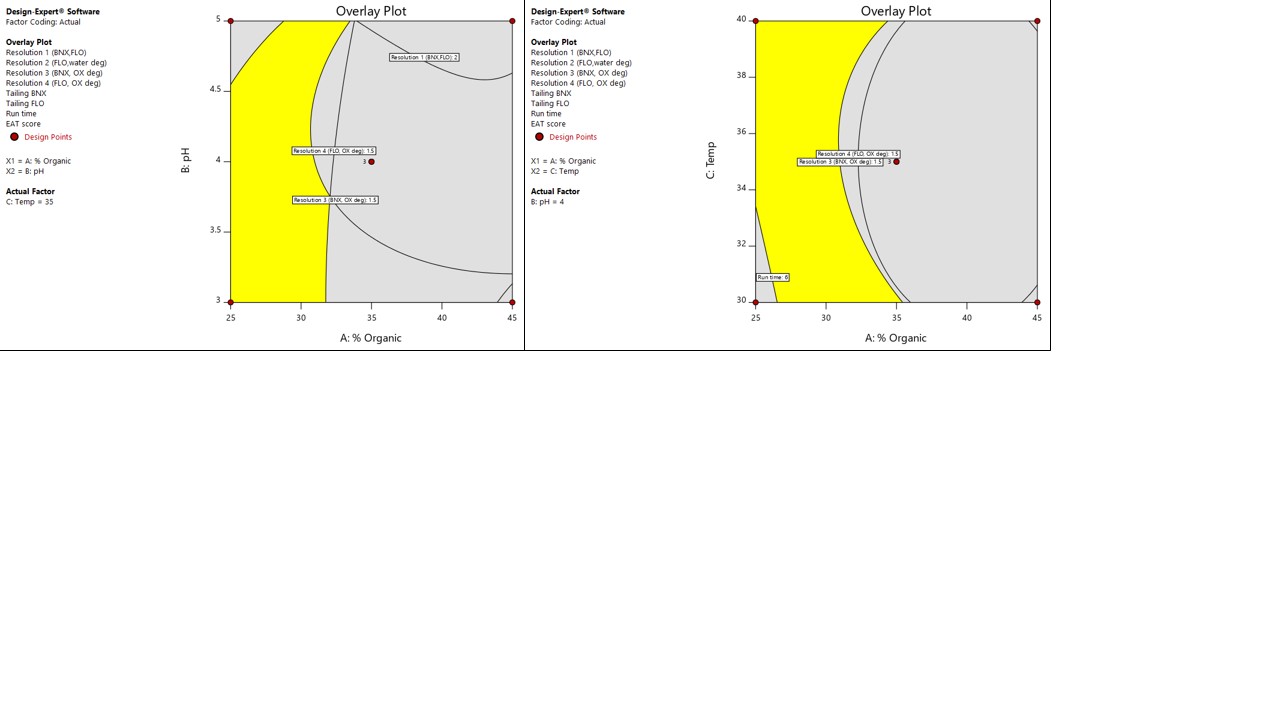


**Supplementary Fig. S4**


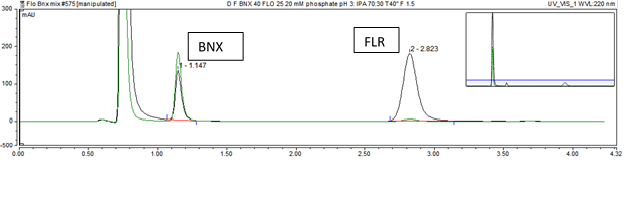


**Supplementary Fig. S5**

Table S1: Fractional factorial design matrix for the effect of different CMPs on CQAs

| Run | A:Buffer conc (mM) | B:TEA | C:pH | D:Organic modifier | E: Ratio of organic (%) | F:Flow rate (mL/min) | G:Column temp | Resolution BNX &FLR | Tailing BNX | Tailing FLR | Run time (minute) | Ecoscale | EAT score |
| --- | --- | --- | --- | --- | --- | --- | --- | --- | --- | --- | --- | --- | --- |
| 1 | 50 | 0.01 | 6 | Isopropanol | 65 | 1.5 | 40 | 2.17 | 1.36 | 1.41 | 1.00 | 82 | 2.73 |
| 2 | 20 | 0.01 | 3 | Ethanol | 65 | 1.5 | 25 | 1.30 | 1.11 | 1.58 | 1.50 | 80 | 2.94 |
| 3 | 20 | 0 | 6 | Isopropanol | 65 | 1.0 | 25 | 1.83 | 1.33 | 1.18 | 1.60 | 90 | 2.70 |
| 4 | 20 | 0 | 3 | Isopropanol | 25 | 1.5 | 40 | 10.44 | 1.18 | 1.14 | 4.50 | 88 | 4.38 |
| 5 | 20 | 0 | 6 | Ethanol | 65 | 1.5 | 40 | 6.10 | 1.14 | 1.30 | 3.50 | 86 | 6.87 |
| 6 | 20 | 0.01 | 3 | Isopropanol | 65 | 1.0 | 40 | 1.45 | 1.31 | 1.47 | 1.40 | 82 | 2.36 |
| 7 | 20 | 0.01 | 6 | Ethanol | 25 | 1.0 | 40 | 4.12 | 1.39 | 1.33 | 11.50 | 78 | 5.78 |
| 8 | 50 | 0.01 | 3 | Isopropanol | 25 | 1.0 | 25 | 16.13 | 1.53 | 1.30 | 17.50 | 80 | 11.37 |
| 9 | 50 | 0 | 3 | Ethanol | 65 | 1.0 | 40 | 1.38 | 1.14 | 1.41 | 1.50 | 88 | 1.96 |
| 10 | 50 | 0.01 | 3 | Ethanol | 25 | 1.5 | 40 | 15.02 | 1.21 | 1.05 | 18.30 | 78 | 13.81 |
| 11 | 50 | 0 | 6 | Isopropanol | 25 | 1.0 | 40 | 7.48 | 1.44 | 1.80 | 3.50 | 90 | 2.27 |
| 12 | 50 | 0 | 6 | Ethanol | 25 | 1.5 | 25 | 4.71 | 1.67 | 1.37 | 11.80 | 86 | 8.90 |
| 13 | 50 | 0 | 3 | Isopropanol | 65 | 1.5 | 25 | 1.73 | 1.37 | 2.03 | 2.70 | 90 | 6.84 |
| 14 | 20 | 0 | 3 | Ethanol | 25 | 1.0 | 25 | 17.00 | 1.71 | 1.60 | 25.00 | 86 | 12.57 |
| 15 | 50 | 0.01 | 6 | Ethanol | 65 | 1.0 | 25 | 5.24 | 1.39 | 1.71 | 2.00 | 80 | 2.62 |
| 16 | 20 | 0.01 | 6 | Isopropanol | 25 | 1.5 | 25 | 4.96 | 1.85 | 1.12 | 2.80 | 82 | 2.73 |

TEA: triethylamine

**Table S2:** Box Behnken design matrix for the effect of different CMPs on CQAs

| Run | A:% Organic | B:pH | C:Temp | Resolution 1 (BNX,FLR) | Resolution 2 (FLR,water deg) | Resolution 3 (BNX, OX deg) | Resolution 4 (FLR, OX deg) | Tailing BNX | Tailing FLR | Run time (minute) | EAT score |
| --- | --- | --- | --- | --- | --- | --- | --- | --- | --- | --- | --- |
| 1 | 35 | 4 | 35 | 5.23 | 4.69 | 0.60 | 0.00 | 1.68 | 1.51 | 3.00 | 4.09 |
| 2 | 35 | 4 | 35 | 5.14 | 4.56 | 0.40 | 0.00 | 1.53 | 1.40 | 2.90 | 3.96 |
| 3 | 35 | 3 | 40 | 5.64 | 5.10 | 4.45 | 2.02 | 1.37 | 1.32 | 2.30 | 3.14 |
| 4 | 45 | 5 | 35 | 1.51 | 1.81 | 0.00 | 0.50 | 1.22 | 1.22 | 1.30 | 2.28 |
| 5 | 35 | 5 | 30 | 3.26 | 2.43 | 0.81 | 2.40 | 1.37 | 1.17 | 1.80 | 2.45 |
| 6 | 45 | 3 | 35 | 3.40 | 3.59 | 1.97 | 1.35 | 1.43 | 1.37 | 2.00 | 3.51 |
| 7 | 25 | 4 | 30 | 14.47 | 3.20 | 5.39 | 11.16 | 1.6 | 1.55 | 7.30 | 7.11 |
| 8 | 35 | 4 | 35 | 5.20 | 4.60 | 0.70 | 0.00 | 1.6 | 1.46 | 3.20 | 4.36 |
| 9 | 25 | 5 | 35 | 5.04 | 0.00 | 4.87 | 10.19 | 1.49 | 1.32 | 3.40 | 3.31 |
| 10 | 45 | 4 | 40 | 1.87 | 2.68 | 0.00 | 1.14 | 1.21 | 1.35 | 1.50 | 2.63 |
| 11 | 35 | 5 | 40 | 2.65 | 2.47 | 1.98 | 2.40 | 1.17 | 1.25 | 1.60 | 2.18 |
| 12 | 25 | 3 | 35 | 14.85 | 3.32 | 4.24 | 6.90 | 1.36 | 1.16 | 5.50 | 5.36 |
| 13 | 45 | 4 | 30 | 2.68 | 3.00 | 0.00 | 1.49 | 1.74 | 1.56 | 2.50 | 4.38 |
| 14 | 35 | 3 | 30 | 6.74 | 5.31 | 4.63 | 1.96 | 1.35 | 1.28 | 2.50 | 3.41 |
| 15 | 25 | 4 | 40 | 13.17 | 2.53 | 3.58 | 10.91 | 1.34 | 1.15 | 4.70 | 4.58 |

**Table S3:** Results of system suitability tests for the proposed HPLC method

| **Parameters** | **BNX HCl** | **FLR sodium** |
| --- | --- | --- |
| Retention time (t_R_ min) | 1.14 ± 0. 09 | 2.85 ± 0.19 |
| Resolution (R_s_) | 6.32 | |
| Theoretical plates (N) | 3198 | 8081 |
| Asymmetry factor | 1.12 | 1.21 |

* Mean ± SD, n = 6.

Table S4: Evaluation of accuracy for the determination of BNX HCl and FLR sodium

| **Drug** | **conc. taken (µg/ml)** | **conc. found (µg/ml)** | **% Recovery** | **Mean % recovery ± SD** |
| --- | --- | --- | --- | --- |
| BNX HCl | 20.00 | 19.90 | 99.52 | 100.04 ± 0.53 |
|  | 40.00 | 40.30 | 100.76 |  |
|  | 60.00 | 59.90 | 99.84 |  |
| FLR sodium | 12.50 | 12.50 | 100.01 | 100.26 ± 0.81 |
|  | 25.00 | 25.29 | 101.17 |  |
|  | 50.00 | 49.80 | 99.61 |  |

Table S5: Evaluation of the precision of the proposed HPLC method for the determination of BNX HCl and FLR sodium

| **Drug** | **Intra day** | | | **Inter day** | | |
| --- | --- | --- | --- | --- | --- | --- |
|  | **conc. taken (µg/ml)** | **conc. found (µg/ml)** | **%RSD** | **conc. taken (µg/ml)** | **conc. found (µg/ml)** | **%RSD** |
| BNX HCl | 20.00 | 19.99 | 0.44 | 20.00 | 19.90 | 0.95 |
|  |  | 19.79 |  |  | 20.07 |  |
|  |  | 19.92 |  |  | 19.62 |  |
|  | 40.00 | 40.31 | 0.43 | 40.00 | 40.17 | 0.79 |
|  |  | 40.28 |  |  | 40.37 |  |
|  |  | 39.93 |  |  | 40.93 |  |
|  | 60.00 | 59.69 | 0.25 | 60.00 | 59.90 | 0.31 |
|  |  | 60.05 |  |  | 59.45 |  |
|  |  | 59.97 |  |  | 59.64 |  |
| FLR sodium | 12.50 | 12.60 | 0.88 | 12.50 | 12.48 | 1.04 |
|  |  | 12.45 |  |  | 12.27 |  |
|  |  | 12.38 |  |  | 12.25 |  |
|  | 25.00 | 25.28 | 0.19 | 25.00 | 25.27 | 0.43 |
|  |  | 25.31 |  |  | 25.16 |  |
|  |  | 25.22 |  |  | 25.05 |  |
|  | 50.00 | 49.98 | 0.41 | 50.00 | 49.78 | 0.58 |
|  |  | 49.57 |  |  | 49.42 |  |
|  |  | 49.80 |  |  | 49.22 |  |

**Table S6:** Robustness results for the proposed HPLC method

| FLR sodium | | | | BNX HCl | | | | Parameters | |
| --- | --- | --- | --- | --- | --- | --- | --- | --- | --- |
| R.S.D | **S.D** | **Mean recovery %** | **Recovery %** | **R.S.D** | **S.D** | **Mean recovery %** | **Recovery %** |  |  |
| 0.60 | 0.60 | 99.71 | 98.94 | 0.89 | 0.89 | 99.94 | 99.09 | 25 | Ratio of isopropanol |
|  |  |  | 99.76 |  |  |  | 99.87 | 27 |  |
|  |  |  | 100.42 |  |  |  | 100.87 | 29 |  |
| 0.71 | 0.71 | 98.78 | 98.13 | 0.68 | 0.68 | 100.59 | 101.23 | 2.9 | pH of aqueous component of mobile phase |
|  |  |  | 99.76 |  |  |  | 99.87 | 3.0 |  |
|  |  |  | 98.45 |  |  |  | 100.69 | 3.1 |  |
| 0.46 | 0.46 | 99.15 | 99.05 | 0.59 | 0.59 | 100.48 | 101.06 | 1.4 | Flow rate |
|  |  |  | 99.76 |  |  |  | 99.87 | 1.5 |  |
|  |  |  | 98.65 |  |  |  | 100.52 | 1.6 |  |
| 0.39 | 0.39 | 99.84 | 99.40 | 0.95 | 0.95 | 100.00 | 99.12 | 38 | Column Temperature |
|  |  |  | 99.76 |  |  |  | 99.87 | 40 |  |
|  |  |  | 100.35 |  |  |  | 101.02 | 42 |  |

Table S7: Application of the proposed method and comparison method for the determination of the studied drugs in laboratory prepared mixture.

| Conc. Taken (µg/mL) | | Proposed method | | Reported method [22] | |
| --- | --- | --- | --- | --- | --- |
|  |  | % Recovery | | % Recovery | |
| BNX HCl | FLR sodium | BNX HCl | FLR sodium | BNX HCl | FLR sodium |
| 40.00 | 25.00 | 99.40 | 100.22 | 99.95 | 98.27 |
|  |  | 98.56 | 100.96 | 101.42 | 99.48 |
|  |  | 98.06 | 99.37 | 100.88 | 99.58 |
|  |  | 99.93 | 99.65 | 99.62 | 98.09 |
|  |  | 99.55 | 99.25 | 100.50 | 98.73 |
|  |  | 99.79 | 99.85 | 100.19 | 99.19 |
| Mean + SD | | 99.21+0.68 | 100.43+0.59 | 99.514+0.852 | 98.89+0.57 |
| t-test | | 1.18 (2.228) | | 0.976 (2.228) | |
| F-test | | 0.77 (5.050) | | 0.98 (5.05) | |

*Values in parenthesis are the tabulated t- and F- values at p=0.05.
